# A putative cAMP-binding protein in *Trypanosoma brucei* cooperates with FLAM3 to promote flagellar connection and cell morphogenesis

**DOI:** 10.1016/j.jbc.2024.107856

**Published:** 2024-10-05

**Authors:** Qing Zhou, Phu Van Nguyen, Ziyin Li

**Affiliations:** Department of Microbiology and Molecular Genetics, McGovern Medical School, University of Texas Health Science Center at Houston, Houston, Texas, USA

**Keywords:** *Trypanosoma brucei*, flagellum, trypomastigote, epimastigote, cAMP-binding protein, flagellum attachment zone

## Abstract

*Trypanosoma brucei* is a flagellated parasitic protozoan, and within the insect vector the parasite transitions from the trypomastigote form to the epimastigote form by repositioning its mitochondrial genome and relocating the flagellum. The mechanisms underlying such morphology changes are still poorly understood, but several flagellum-localized proteins are involved in this process by modulating the flagellum attachment zone (FAZ) that adheres the flagellum to the cell membrane. We report here a putative cAMP-binding protein named cAMP-BP1, which promotes flagellar connection and morphology transition. cAMP-BP1 contains two cyclic nucleotide-binding domains and five calcium-binding C2 domains and localizes to the flagella connector and the new FAZ tip. Depletion of cAMP-BP1 in the trypomastigote form of *T. brucei* causes major morphology changes, generating epimastigote-like cells with repositioned kinetoplast and relocated flagellum. At the flagella connector and the new FAZ tip, cAMP-BP1 associates with FLAM3, a regulator of morphology transition, depends on the latter for localization, and is required for FLAM3 localization to the flagella connector. Knockdown of cAMP-BP1 inhibits FAZ elongation and disrupts flagellar connection by impairing flagella connector structural integrity. These results identify a flagella connector- and new FAZ tip-localized protein as a regulator of morphology transition and flagellar connection in trypanosomes and uncover its functional interplay with FLAM3 to promote FAZ elongation for maintaining trypomastigote morphology.

*Trypanosoma brucei* is a unicellular protozoan parasite and the causative agent of sleeping sickness in humans and nagana in cattle in sub-Saharan Africa. The parasite has a single flagellum, which is involved in cell motility, cell morphogenesis, cell division, and cell-cell communications. The flagellum is nucleated from the basal body, a centriole-like structure located at the posterior portion of the cell, exits the cell body through the flagellar pocket, attaches to the cell membrane *via* a specialized cytoskeletal structure termed the flagellum attachment zone (FAZ), and extends toward the cell anterior (1). During the S phase of the cell cycle, a new flagellum is assembled next to the old flagellum, and its distal tip is tethered to the lateral side of the old flagellum in the insect (procyclic) form of *T. brucei via* a three-layered transmembrane junction termed the flagella connector (FC), a dynamic and mobile structure that was proposed to be implicated in the inheritance of cell pattern (2, 3, 4). The FC is formed concomitant with the assembly of the new flagellum, persists during the elongation of the new flagellum during the cell cycle, and finally is removed when cytokinesis is initiated (3). In the bloodstream form of *T. brucei*, however, an FC-like structure is not present and, instead, the distal tip of the new flagellum is embedded, besides the old flagellum, in a discrete indentation of the cell membrane termed the groove, a mobile transmembrane junction that migrates while the new flagellum elongates and resolves upon cytokinesis initiation (5).

Structurally, the FC is composed of five distinct subdomains between the membrane junction of the new and the old flagella. The FC zone 1, represented by the FCP4 protein, is located at the distal tip of the new flagellum and contains a filamentous network connecting the axonemal microtubules to the FC zone 2, an electron-dense layer lying underneath the membrane of the new flagellum at the junction. No representative protein for zone 2 has been identified so far. The FC zone 3, represented by FC1 and FCP1, is an electron-dense layer between the membrane of the new and the old flagella at the junction. The FC zone 4, represented by FCP3, is an electron-dense layer underneath the membrane of the old flagellum, which connects the FC zone 5 that is represented by FCP2 and comprises a filamentous network linked to the axonemal microtubules in the old flagellum (4, 6). Depletion of individual FC components results in premature disconnection of the new flagellum from the old flagellum, demonstrating the essential role for the FC in mediating flagellar connection in the procyclic form (4, 7).

The FAZ in *T. brucei* is a complex cytoskeletal structure comprising discrete subdomains at the intermembrane junction between the flagellum and the cell body and plays crucial roles in flagellum attachment, cell morphogenesis, and cell division (8). Within the flagellum, the FAZ contains a so-called FAZ flagellum domain that links the flagellar membrane to the paraflagellar rod (PFR) and the flagellar axonemal microtubules and, within the cell body, the FAZ contains an intracellular FAZ filament and other structures that link the cell membrane to the FAZ filament and its associated quartet microtubules (8). Assembly of the FAZ is initiated during the S-phase of the cell cycle, and new components are incorporated into the elongating FAZ from its proximal end in a coordinated manner with the elongation of the flagellum (9, 10).

*T. brucei* has a complex life cycle by alternating between the insect vector, the tsetse fly, and the mammalian hosts, and it transitions to morphologically distinct life cycle forms during its life cycle development. These life cycle forms are distinguished by the position of the cell’s mitochondrial DNA complex termed the kinetoplast, the position of the flagellum, and the length of the unattached flagellum (11). In the fly's gut, *T. brucei* transitions from the trypomastigote form to the epimastigote form by repositioning its kinetoplast and flagellum from the posterior side of the nucleus to the anterior side. The epimastigote form proliferates through asymmetrical cell division and then migrates to the salivary gland where it further differentiates into the mammal-infective metacyclic form (11). The mechanisms underlying life cycle form transitions are poorly understood, but the discoveries that three FAZ flagellum domain proteins, ClpGM6, FLAM3, and FAZ27 (12, 13, 14), and two intracellular FAZ filament proteins, FAZ9 (7) and TbSAS-4 (15), are involved in the trypomastigote-to-epimastigote transition suggest the requirement of the modulation of FAZ structure for morphology transitions.

We report here a novel cyclic nucleotide-binding (CNB) domain- and C2 domain-containing protein that localizes to the FC and the new FAZ tip and is required for cell morphogenesis, by modulating the FAZ, and for flagellar connection, by maintaining FC integrity. This protein, named cAMP-BP1, is the first protein found to localize to the FC and the new FAZ tip and play essential roles in cell morphogenesis and flagellar connection in trypanosomes. cAMP-BP1 associates with FLAM3, depends on FLAM3 for localization, and is require for FLAM3 localization to the FC. cAMP-BP1 also depends on the two putative CNB domains and one of the five C2 domains for its cellular function. These findings highlight the unique contribution of a FLAM3-associated protein at the FC and the new FAZ tip to morphology transition and flagellar connection, and imply the potential involvement of cAMP in regulating these processes in trypanosomes.

## Results

### A putative cAMP-binding protein localizes to the FC and the new FAZ tip

**Figure 1:**
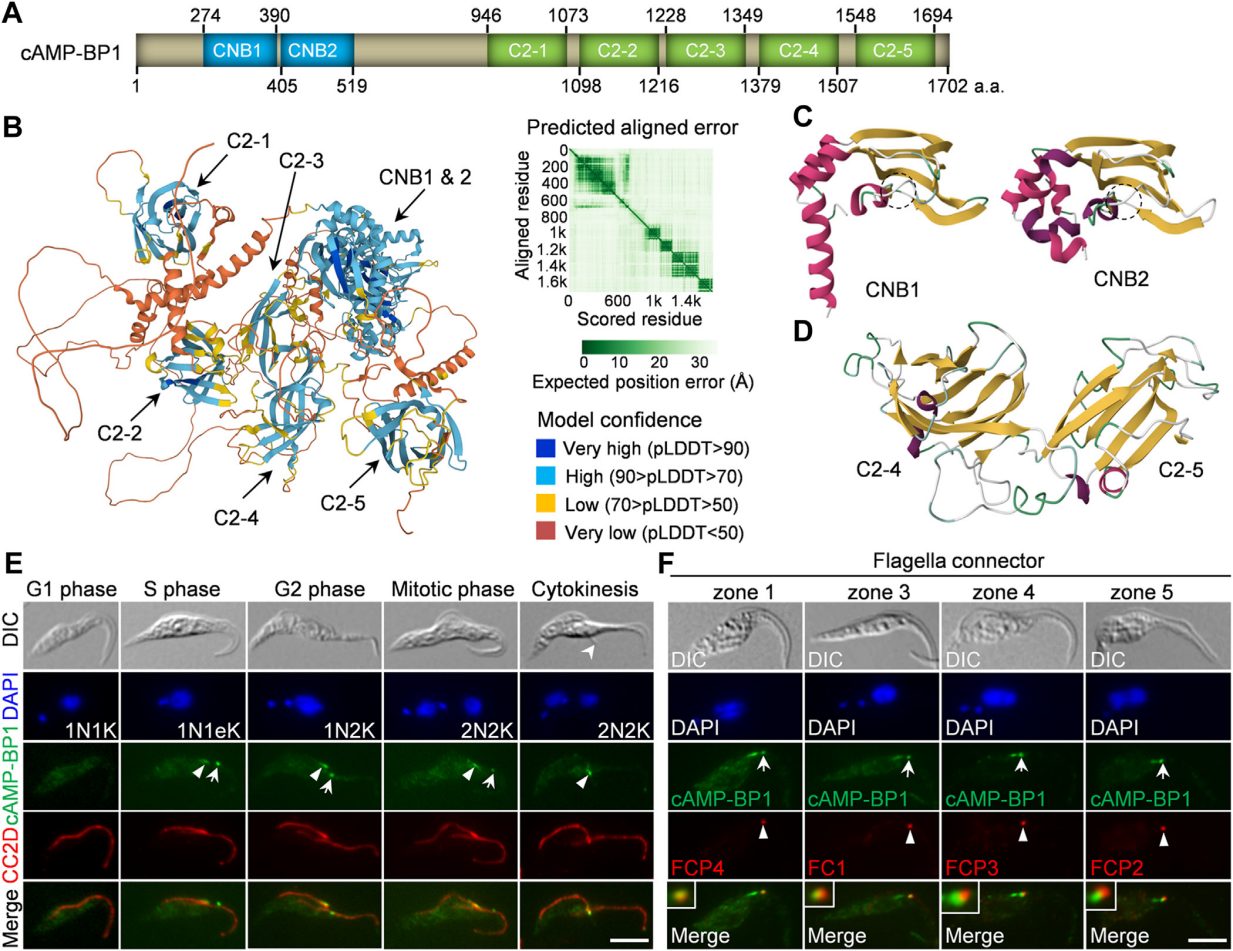
**A CNB domain- and C2 domain-containing protein localizes to the flagella connector and the new FAZ tip.***A*, schematic illustration of the structural motifs in cAMP-BP1. CNB: cyclic nucleotide binding domain; C2: C2 domain. *B*, predicted structure of cAMP-BP1 by AlphaFold. The structural domains are indicated. *C*, homology modeling of the two CNB domains in cAMP-BP1, using templates 5j3u.1.A (*Toxoplasma gondii* PKA) and 6hyq.2.A (*Trypanosoma cruzi* PKA), respectively. The black *dotted-line circle* indicates the predicted cAMP-binding site. *D*, homology modeling of the last two C2 domains (C2-4 and C2-5) in cAMP-BP1, using the template 4npj.1.A (Extended-Synaptotagmin 2, C2A- and C2B-domains). *E*, subcellular localization of cAMP-BP1 during the cell cycle. Endogenously expressed cAMP-BP1-3HA was coimmunostained with anti-CC2D antibody to label the FAZ filament. *Solid arrowheads* indicate cAMP-BP1 signal at the new FAZ tip, and *arrows* indicate cAMP-BP1 signal at the FC. The *open arrowhead* in the DIC image indicates the tip of the detached new flagellum. The scale bar represents 5 μm. *F*, coimmunostaining of cells expressing cAMP-BP1-3HA and PTP-tagged FC proteins from different zones of the FC structure. *Arrows* indicate cAMP-BP1 signal at the FC, and *arrowheads* indicate the FC proteins. Insets show the zoom-in view of the colocalization of cAMP-BP1 and FC proteins at the flagella connector. The scale bar represents 5 μm. CNB, cyclic nucleotide-binding; DIC, differential interference contrast; FAZ, flagellum attachment zone; FC, flagella connector; PKA, protein kinase A.

We attempted to identify cytokinesis regulatory proteins by localization-based screening for new FAZ tip-localized proteins from the list of S-phase-enriched proteins identified previously by the cell cycle-regulated transcriptome analysis (16). We discovered a putative cAMP-binding protein (TriTrypDB accession number: Tb927.10.5240) that localizes to the new FAZ tip and the FC in the procyclic form of *T. brucei* (see Fig. 1 below). We named this protein cAMP-BP1 for cAMP-binding protein 1 based on the TriTrypDB annotation. Structural modeling by SWISS-MODEL (17) detected two putative CNB domains and two putative C2 domains, and structural prediction by AlphaFold (18, 19) (https://colab.research.google.com/github/sokrypton/ColabFold/blob/main/AlphaFold2.ipynb#scrollTo=kOblAo-xetgx) detected three additional C2 domains (Fig. 1, *A* and *B*). The two CNB domains are arranged in tandem at the N terminus, each of which comprises several α-helices and an eight-stranded antiparallel β-sandwich (Fig. 1*C*), and the five C2 domains are located at the C terminus, each of which is composed of an eight-stranded β-sandwich (Fig. 1*D*). The CNB domain, found in the cAMP-dependent protein kinase A, the cGMP-dependent protein kinase G, and other cAMP-related regulatory proteins (20), binds cyclic nucleotide phosphate through the conserved phosphate-binding cassette comprising of β-strand 6, a short α-helix, and β-strand 7 of the β-sandwich (20). Within the phosphate-binding cassette, a conserved glutamate residue binds the ribose 2′-OH of cAMP and a conserved arginine residue binds the exocyclic phosphate of cAMP (20). Both the predicted CNB domains, CNB1 and CNB2, adopt the typical fold of the CNB domain found in the known cAMP-binding proteins (Figs. 1*C* and S1*A*). CNB1 contains the conserved glutamate and arginine residues required for cAMP binding, whereas CNB2 lacks the conserved arginine residue (Fig. S1*B*). Close homologs for cAMP-BP1 are found in all of the kinetoplastid organisms with available genome sequences, indicating its conservation in this group of organisms.

We investigated the subcellular localization of cAMP-BP1 in the procyclic form of *T. brucei* by endogenously tagging cAMP-BP1 with a C-terminal triple hemagglutinin (HA) epitope and then by coimmunofluorescence microscopy with certain cytoskeletal markers. cAMP-BP1 was detectable at two distinct foci, one of which overlapped with the distal tip of the newly assembled FAZ from S-phase to cytokinesis and the other of which appeared to associate with the FC (Fig. 1*E*). The cAMP-BP1 focus on the FC emerged from S-phase, but disappeared upon the disconnection of the new flagellum from the old flagellum when cytokinesis is initiated (Fig. 1*E*). To determine the precise location of cAMP-BP1 within the FC, we coimmunostained endogenously 3HA-tagged cAMP-BP1 with the FC proteins from different FC subdomains, FCP4 from zone 1, FC1 from zone 3, FCP3 from zone 4, and FCP2 from zone 5, each of which was endogenously tagged with a C-terminal PTP epitope (Fig. 1*F*). The results showed that cAMP-BP1 overlapped almost completely with FCP4 and was proximal to FC1, FCP3, and FCP2 (Fig. 1*F*), suggesting that cAMP-BP1 is located in the FC zone 1 within the new flagellum. Thus, cAMP-BP1 has a dual localization at the FC and the new FAZ tip.

### Knockdown of cAMP-BP1 by RNAi causes major changes in cell morphology

**Figure 2:**
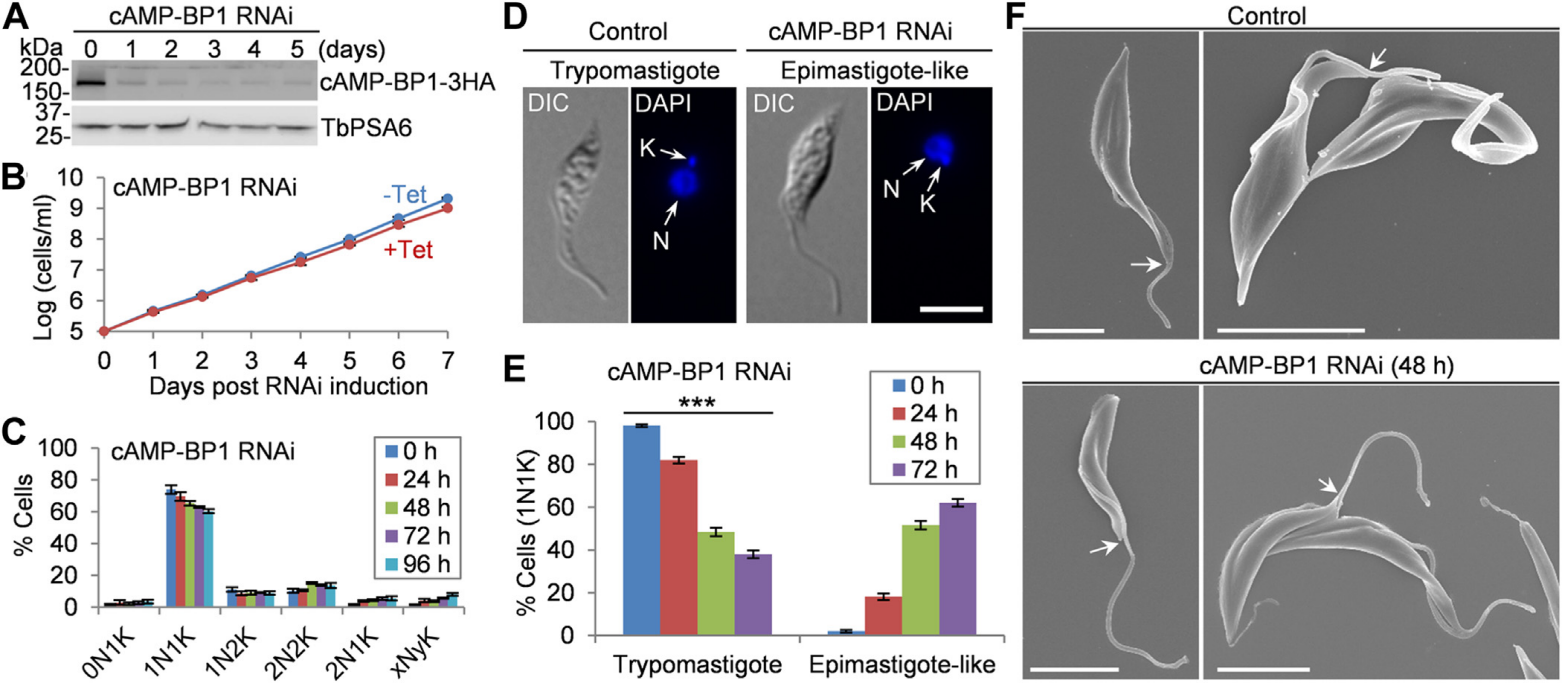
**RNAi-mediated knockdown of cAMP-BP1 causes a major cell morphology change**. *A*, Western blotting to detect the cAMP-BP1 level before and after cAMP-BP1 RNAi induction. cAMP-BP1 was endogenously tagged with a triple HA epitope and detected by the anti-HA antibody. TbPSA6 served as a loading control. *B*, effect of cAMP-BP1 RNAi on cell proliferation. *C*, effect of cAMP-BP1 knockdown on cell cycle progression. Shown is the counting of cells with different numbers of kinetoplasts (K) and nuclei (N) after RNAi induction for up to 96 h. Error bars indicate S.D. from three independent experiments. *D*, cell morphology and positioning of the kinetoplast in control and cAMP-BP1 RNAi-induced cells examined under a light microscope. N, nucleus; K, kinetoplast. The scale bars represent 5 μm. *E*, percentages of 1N1K cells of trypomastigote and epimastigote-like morphology in control and cAMP-BP1 RNAi cells induced for up to 72 h. Error bars indicated S.D. from three independent experiments. ∗∗∗*p* < 0.001. *F*, cell morphology of control and cAMP-BP1 RNAi-induced cells examined by scanning electron microscopy. *Arrows* indicate the distal end of the cell body (uniflagellated cells) and the distal end of the cell body of the new-flagellum daughter cell (biflagellated cells). The scale bars represent 5 μm. HA, hemagglutinin; RNAi, RNA interference.

Previously, several new FAZ tip-localized proteins were found to play essential roles in cytokinesis (7, 21, 22, 23, 24, 25, 26, 27, 28), and several FC-localized proteins were reported to control flagellar connection (4, 7). The dual localization of cAMP-BP1 to the new FAZ tip and the FC led us to hypothesize that cAMP-BP1 may regulate both cytokinesis and flagellar connection. To test this possibility, we carried out RNA interference (RNAi) in the procyclic form of *T. brucei*. Induction of RNAi caused a decrease in the levels of cAMP-BP1, which was endogenously tagged with a triple HA epitope, from 1 day of RNAi induction, but it did not completely deplete the protein even after 5 days (Fig. 2*A*). This downregulation of cAMP-BP1 caused weak growth defects after 4 days of RNAi induction (Fig. 2*B*) and a slight change in cell cycle progression, producing small numbers of abnormal cell types, such as 0N1K cells (∼3%), 2N1K cells (∼5%), and xNyK (x > 2, y ≥ 1) cells (∼8%) (Fig. 2*C*). Thus, although cAMP-BP1 is localized to the new FAZ tip, it is not an essential regulator of cytokinesis, unlike those new FAZ tip-localized cytokinesis regulators, such as CIF1-CIF4 and FPRC, which are essential for cytokinesis (21, 22, 23, 25, 26).

Knockdown of cAMP-BP1 caused a major change in cell morphology, resulting in the production of epimastigote-like cells to ∼60% of the 1N1K cell population after 72 h of RNAi induction (Fig. 2, *D* and *E*). The characteristic features of the epimastigote-like morphology of trypanosome cells include a long, free (unattached) flagellum and a repositioned kinetoplast, which is either juxtaposed or anterior to the nucleus, in contrast to the trypomastigote morphology in which the kinetoplast is located posterior to the nucleus (Fig. 2*D*). Scanning electron microscopy further confirmed the epimastigote-like morphology of the cells with a single flagellum after cAMP-BP1 RNAi (Fig. 2*F*). The biflagellated cells possessed a long, free new flagellum and a normal old flagellum (Fig. 2*F*), which, presumably, will produce an epimastigote-like daughter cell and a normal-shaped daughter cell after cell division. Notably, some biflagellated cells had long unattached new and old flagella (Fig. S2*A*). Such cells were likely to be derived from the epimastigote-like 1N1K cells following an additional round of cell cycle. This type of biflagellated cells further accumulated after prolonged RNAi induction and reached ∼67% of the total 2N2K population after 48 h of RNAi induction (Fig. S2*B*).

### Knockdown of cAMP-BP1 disrupts the elongation of the new FAZ

**Figure 3:**
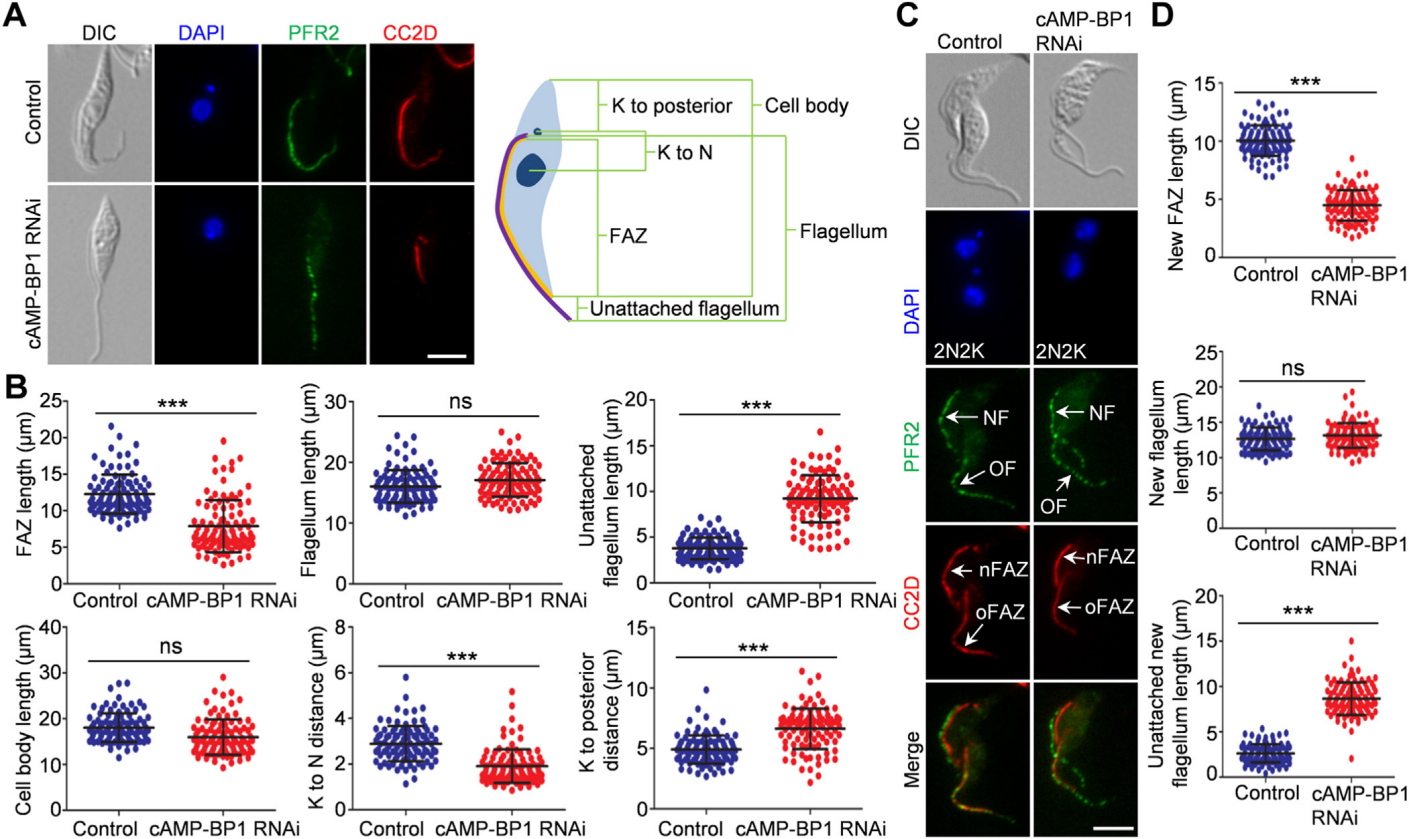
**Knockdown of cAMP-BP1 disrupts FAZ elongation and kinetoplast positioning.***A*, morphology of 1N1K cells collected from control and cAMP-BP1 RNAi-induced population. 1N1K cells were coimmunostained with the anti-CC2D antibody to label the FAZ filament and the anti-PFR2 antibody to label the flagellum. The scale bar represents 5 μm. The cartoon on the *right* panel depicted a 1N1K cell used for measuring the length of the cell body, the flagellum, the unattached flagellum, the FAZ filament, the distance from the kinetoplast (K) to the posterior cell tip, and the distance from the kinetoplast to the nucleus (N). *B*, morphometric measurement of 1N1K cells from control and cAMP-BP1 RNAi-induced (24 h) population. The length of cell body, FAZ, flagellum, unattached flagellum, kinetoplast to nucleus, and kinetoplast to the posterior cell tip were measured and plotted (*n* = 100). ∗∗∗: *p* < 0.001; ns: no significance. *C*, morphology of 2N2K cells collected from control and cAMP-BP1 RNAi-induced population. Cells were coimmunostained with anti-CC2D and anti-PFR2 antibodies to label the FAZ filament and the flagellum, respectively. NF: new flagellum; OF: old flagellum; nFAZ: new FAZ; oFAZ: old FAZ. The scale bar represents 5 μm. *D*, morphometric measurement of 2N2K cells collected from control and cAMP-BP1 RNAi-induced population. The length of the new FAZ filament, the new flagellum, and the unattached new flagellum was measured and plotted (*n* = 100). ∗∗∗: *p* < 0.001; ns: no significance. FAZ, flagellum attachment zone; PFR, paraflagellar rod; RNAi, RNA interference.

Since the epimastigote-like 1N1K cells produced by cAMP-BP1 RNAi possess a long unattached flagellum, we asked whether the elongation of the FAZ was affected by cAMP-BP1 knockdown. We performed coimmunofluorescence microscopy with the anti-CC2D antibody to label the intracellular FAZ filament and the anti-PFR2 antibody to label the flagellum, and measured the FAZ length, the flagellum length, the free flagellum length, the cell body length, the kinetoplast-to-nucleus distance, and the kinetoplast-to-posterior distance of the 1N1K cells (Fig. 3*A*). Knockdown of cAMP-BP1 resulted in a ∼36% reduction of the FAZ length from an average of ∼12.3 μm to ∼7.9 μm (Fig. 3*B*), but it exerted no effect on the length of the flagellum (Fig. 3*B*). Consequently, this resulted in a ∼144% increase of the length of the free flagellum from an average of ∼3.8 μm to ∼9.2 μm (Fig. 3*B*). cAMP-BP1 knockdown caused a slight but insignificant decrease in the cell body length (Fig. 3*B*). Knockdown of cAMP-BP1 resulted in a ∼34% reduction of the kinetoplast-to-nucleus distance from an average of ∼2.9 μm to ∼1.9 μm, and a corresponding increase by ∼35% of the kinetoplast-to-posterior distance from an average of ∼4.9 μm to ∼6.6 μm (Fig. 3*B*). Such morphological changes of the 1N1K cells were also observed in the new-flagellum daughter cell of the 2N2K cells (Fig. 3*C*). Knockdown of cAMP-BP1 caused a ∼55% reduction of the average length of the new FAZ from ∼10 μm to ∼4.5 μm and a corresponding increase of the average length of the free new flagellum by ∼231% from ∼2.6 μm to ∼8.7 μm, without affecting the length of the new flagellum (Fig. 3*D*). Together, these results suggest that cAMP-BP1 knockdown inhibited the elongation of the new FAZ and caused mispositioning of the flagellum and the kinetoplast, generating cells with an epimastigote-like morphology.

### cAMP-BP1 is required for flagellar connection

**Figure 4:**
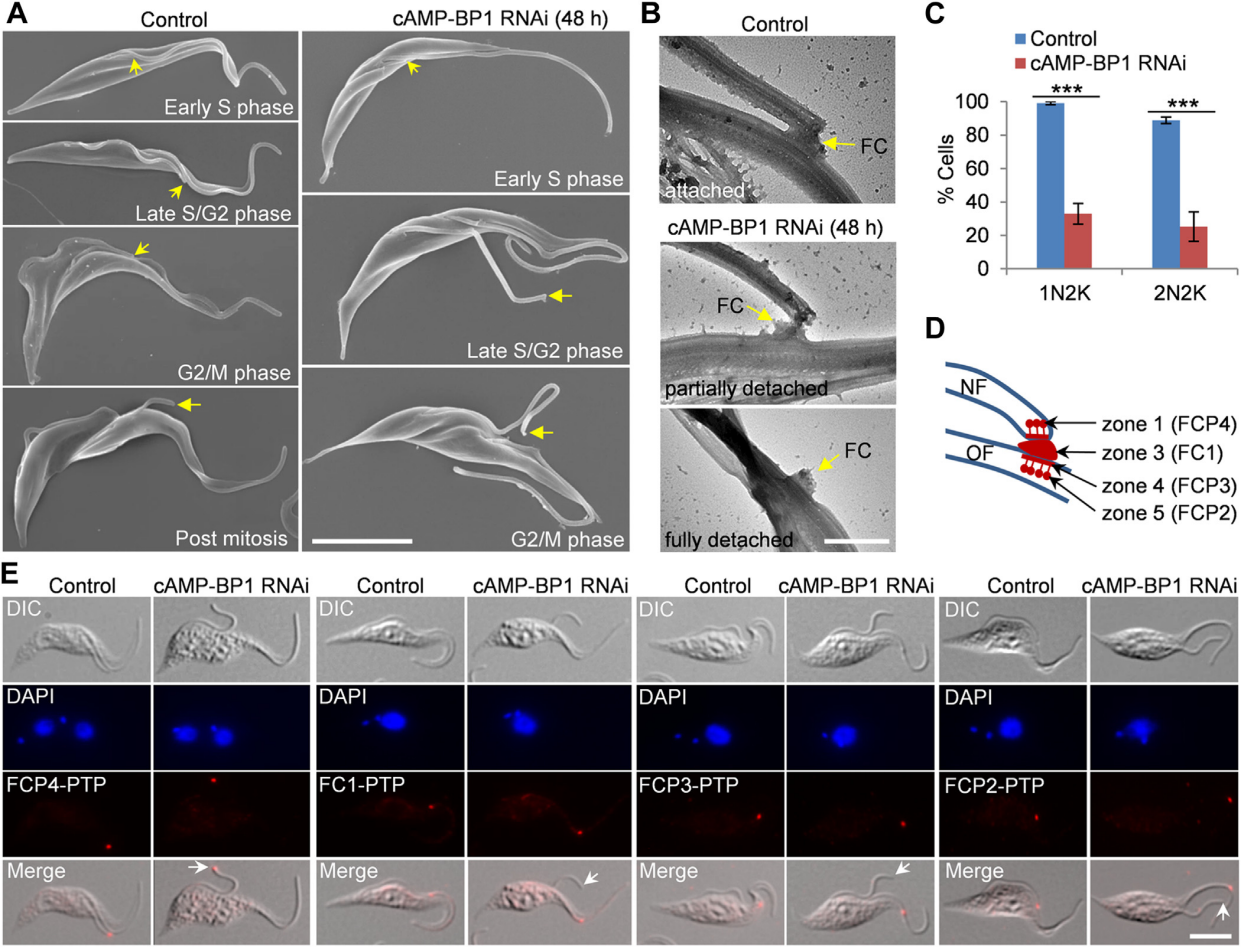
**cAMP-BP1 knockdown disrupts flagellar connection.***A*, examination of flagellar connection in control and cAMP-BP1 RNAi-induced cells by scanning electron microscopy. The *yellow arrows* indicate the distal tip of the new flagellum. The scale bar represents 5 μm. *B*, transmission electron microscopic analysis of the FC in control and cAMP-BP1 RNAi cells. FC: flagella connector. The scale bar represents 500 nm. *C*, quantitation of 1N2K and 2N2K cells with or without flagellar connection before and after cAMP-BP1 RNAi for 48 h. Error bars indicate S.D. from three independent experiments. ∗∗∗*p* < 0.001. *D*, schematic drawing of the subdomains of the FC with representative FC proteins from each FC zone. NF: new flagellum; OF: old flagellum. *E*, effect of cAMP-BP1 knockdown on the localization of FC proteins from different FC subdomains, which were endogenously tagged with a PTP epitope and detected by the anti-protein A antibody. The *white arrows* indicate the distal tip of the detached new flagellum. The scale bar represents 5 μm. RNAi, RNA interference.

The localization of cAMP-BP1 to the FC prompted us to investigate whether it is required for flagellar connection, like those previously characterized FC proteins (4, 7). To this end, we performed scanning electron microscopy to examine flagellar connection before and after cAMP-BP1 RNAi induction. In the noninduced control cells, whenever the new flagellum was clearly visible, it was found to be always tethered to the lateral side of the old flagellum, except when the cells had initiated cytokinesis, in which the tip of the new flagellum was disconnected from the old flagellum (Fig. 4*A*). In cAMP-BP1 RNAi-induced cells, when the new flagellum was short, which represented cells at early S-phase, it was always tethered to the old flagellum (Fig. 4*A*). However, when the new flagellum was longer, which represented cells at late S-phase and beyond, its tip was disconnected from the old flagellum in most of the cells examined (Fig. 4*A*). Using transmission electron microscopy (TEM), we examined the FC of detergent-extracted cytoskeletons in control and cAMP-BP1 RNAi cells, and observed partially severed and fully severed FC in cAMP-BP1 RNAi cells (Fig. 4*B*). In the latter case, the FC remained on the lateral side of the old flagellum (Fig. 4*B*). Quantitatively, ∼66% of the 1N2K cells and ∼64% of the 2N2K cells lost flagellar connection after cAMP-BP1 RNAi induction for 48 h (Fig. 4*C*).

To characterize the effect of cAMP-BP1 knockdown on the integrity of the FC, we epitope-tagged four FC proteins, each of which represents a discrete subdomain of the FC in trypanosomes (4), in the cAMP-BP1 RNAi cell line. FCP4 represents FC zone 1, FC1 represents FC zone 3, FCP3 represents FC zone 4, and FCP2 represents FC zone 5 (Fig. 4*D*). Immunofluorescence microscopy showed that in the 1N2K cells collected from the cAMP-BP1 RNAi-induced population, FCP4 was detected at the distal tip of the detached new flagellum, whereas FC1, FCP3, and FCP2 were all detected on the lateral side of the old flagellum (Fig. 4*E*), in agreement with the observation that the FC remained on the old flagellum (Fig. 4*B*). Thus, cAMP-BP1 knockdown caused premature severing of the connection between zone 1 (or zone 2) and zone 3 of the FC structure, demonstrating that cAMP-BP1 is required for flagellar connection.

### cAMP-BP1 associates with FLAM3 and depends on FLAM3 for localization

**Figure 5:**
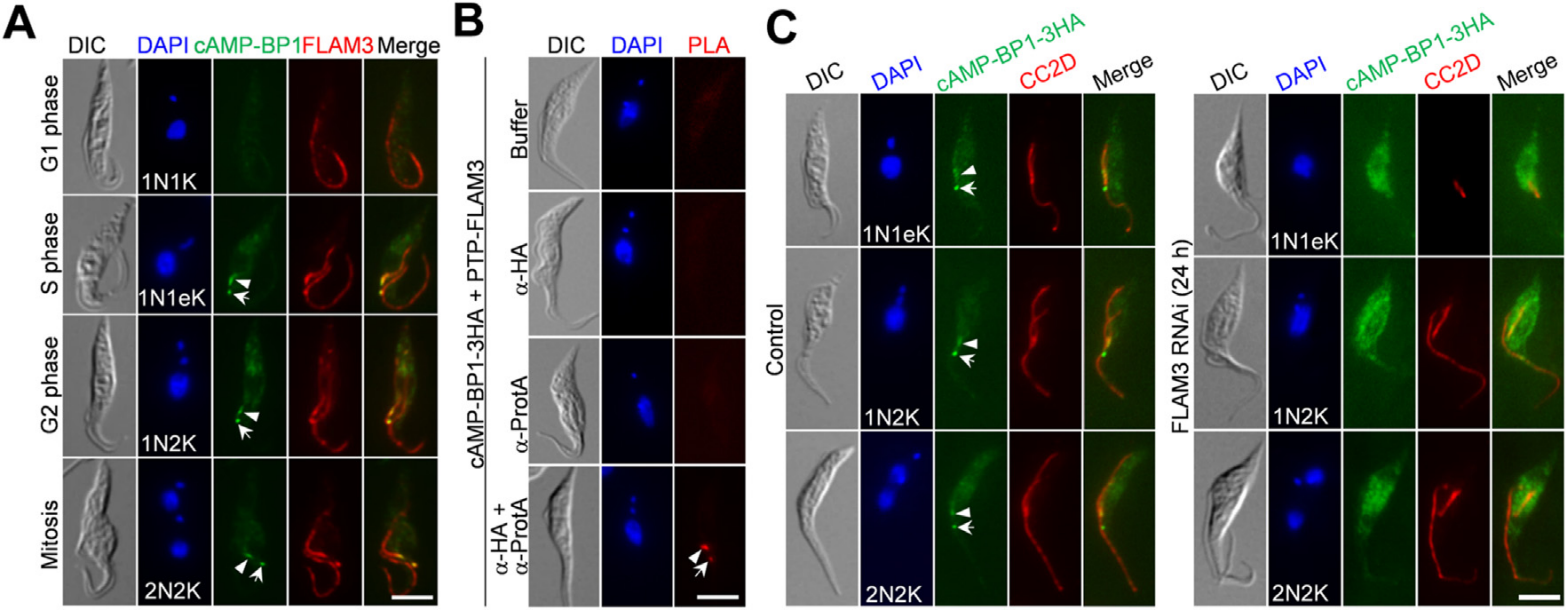
**Localization of cAMP-BP1 to the FC and the new FAZ tip depends on FLAM3**. *A*, coimmunostaining of cells expressing cAMP-BP1-3HA and PTP-FLAM3. *Arrowheads* and *arrow*s indicate cAMP-BP1 signal at the new FAZ tip and the FC, respectively. The scale bar represents 5 μm. *B*, proximity-ligation assay to test the *in vivo* interaction between cAMP-BP1 and FLAM3. *Arrowhead* and *arrow* indicate the positive signal at the new FAZ tip and the FC, respectively. The scale bar represents 5 μm. *C*, effect of FLAM3 knockdown on cAMP-BP1 localization. cAMP-BP1 was endogenously tagged with a tripe HA epitope in the FLAM3 RNAi cell line and detected by the FITC-conjugated anti-HA antibody. The FAZ filament was labeled with the anti-CC2D antibody. *Arrowheads* and *arrow*s indicate cAMP-BP1 signal at the new FAZ tip and the FC, respectively. The scale bar represents 5 μm. FAZ, flagellum attachment zone; FC, flagella connector; RNAi, RNA interference.

FLAM3, a known regulator of cell morphogenesis in *T. brucei*, is enriched in the FC and the FAZ flagellum domain (13, 29). We wondered whether it colocalizes with cAMP-BP1 in the FC and cooperates with cAMP-BP1 to regulate cell morphogenesis. We first expressed cAMP-BP1-3HA and PTP-FLAM3 from their respective endogenous locus in the same cell line and performed coimmunofluorescence microscopy. As reported previously (13, 29), PTP-FLAM3 localized to the FAZ flagellum domain of both the new and the old flagella and the FC (Fig. 5*A*). During the cell cycle stages from S phase to mitosis, cAMP-BP1 colocalized almost completely with FLAM3 at the FC and the distal tip of the FAZ flagellum domain (Fig. 5*A*). To test whether cAMP-BP1 and FLAM3 associate *in vivo* at the FC and/or the new FAZ tip, we carried out proximity ligation assay (PLA), and the results showed positive PLA signal at the FC and the distal tip of new FAZ (Fig. 5*B*), suggesting that cAMP-BP1 and FLAM3 located in such a close proximity that they may form a complex at these locations.

We next investigated whether FLAM3 is required for cAMP-BP1 localization by tagging cAMP-BP1 with a triple HA epitope at its endogenous locus in the FLAM3 RNAi cell line. FLAM3 RNAi was induced for 24 h, and we focused on cells in which the new flagellum was still tethered to the old flagellum (Fig. 5*C*). Knockdown of FLAM3 caused an inhibition of FAZ elongation and produced epimastigote-like cells (Fig. 5*C*), similar to the previous report (13). In all (>1000) of FLAM3 RNAi cells examined, cAMP-BP1 was no longer detectable at the FC and the new FAZ tip (Fig. 5*C*), demonstrating that FLAM3 is required for cAMP-BP1 localization to both structures. Western blotting showed that cAMP-BP1 protein level was not changed (Fig. S3), suggesting that cAMP-BP1 was not degraded, but delocalized.

### Effect of cAMP-BP1 knockdown on the localization of FLAM3 and other FAZ flagellum domain proteins

**Figure 6:**
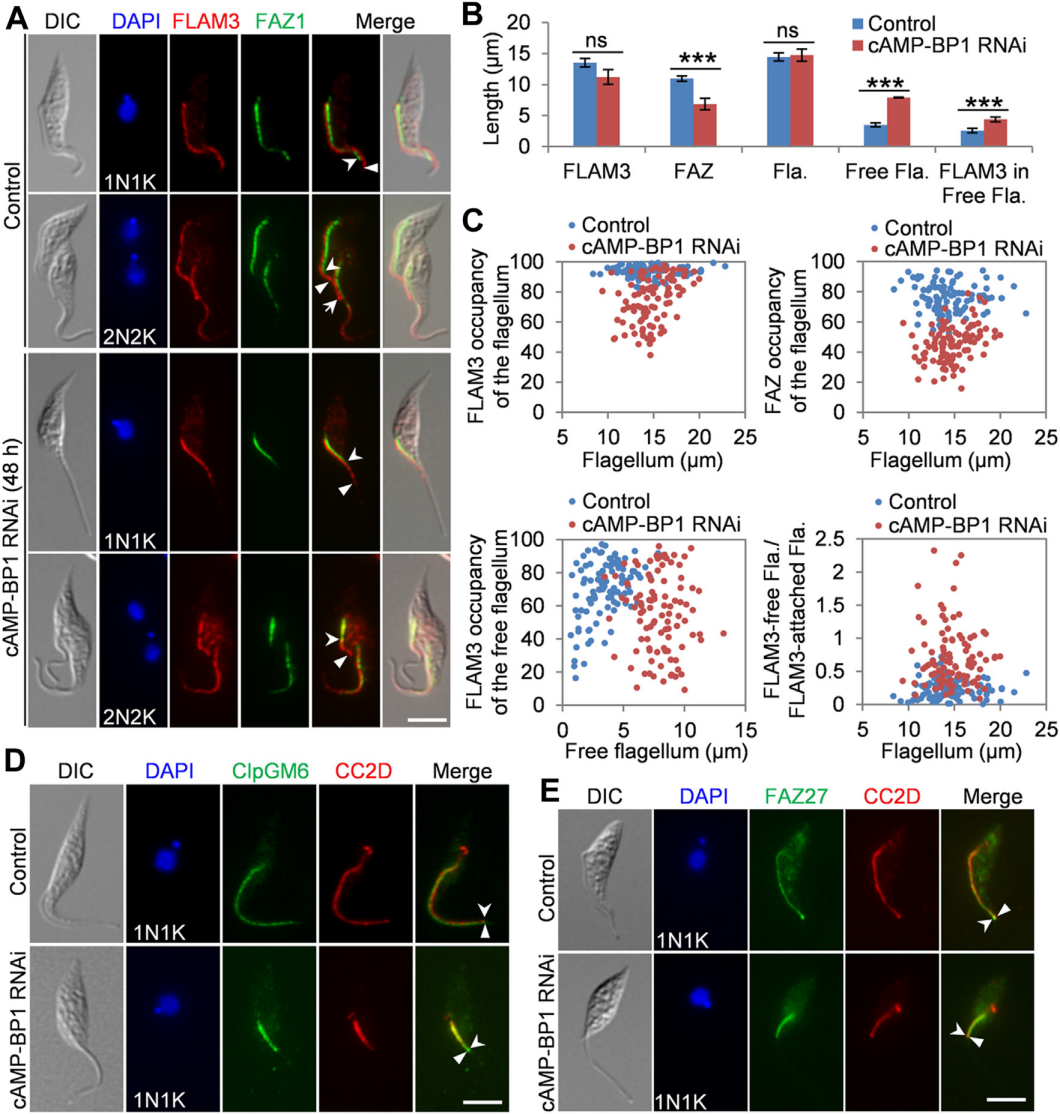
**Knockdown of cAMP-BP1 exerts distinct effect on the FAZ flagellum domain proteins**. *A*, effect of cAMP-BP1 knockdown on the localization of FLAM3. FLAM3 was endogenously tagged with an N-terminal PTP epitope and detected with the anti-protein A antibody. The FAZ filament was labeled with the anti-FAZ1 antibody. *Solid arrowheads* indicate the distal end of the FLAM3 signal within the flagellum, and *open arrowheads* indicate the distal end of the FAZ filament. The *arrow* indicates FLAM3 at the flagella connector. The scale bar represents 5 μm. *B*, the average length of the FLAM3 signal in the flagellum, the FAZ, the flagellum (Fla.), the free flagellum (Fla.), and the FLAM3 signal in the free flagellum (Fla.) of 1N1K cells collected from control and cAMP-BP1 RNAi-induced cell population was calculated and plotted. Error bars indicate S.D. from three independent experiments. ∗∗∗*p* < 0.001; ns: no significance. *C*, the percentage of FLAM3 occupancy of the flagellum, FAZ occupancy of the flagellum, FLAM3 occupancy of the free flagellum, and the ratio of FLAM3 in the free flagellum and FLAM3 in the attached part of the flagellum from control and cAMP-BP1 RNAi cells were plotted based on their measured length. *D*, effect of cAMP-BP1 knockdown of ClpGM6 localization. ClpGM6 was endogenously tagged with an N-terminal triple HA epitope and detected with the FITC-conjugated anti-HA antibody. The FAZ filament was labeled with the anti-CC2D antibody. *Solid arrowheads* indicate the distal end of the ClpGM6 signal, and *open arrowheads* indicate the distal end of the FAZ filament. The scale bar represents 5 μm. *E*, effect of cAMP-BP1 knockdown on the localization of FAZ27. FAZ27 was endogenously tagged with a C-terminal triple HA epitope and detected with the FITC-conjugated anti-HA antibody. The FAZ filament was labeled with the anti-CC2D antibody. *Solid arrowheads* indicate the distal end of the FAZ27 signal, and *open arrowheads* indicate the distal end of the FAZ filament. The scale bar represents 5 μm. FAZ, flagellum attachment zone; RNAi, RNA interference.

We next investigated the effect of cAMP-BP1 depletion on FLAM3 localization by tagging FLAM3 with an N-terminal PTP epitope at its endogenous locus in the cAMP-BP1 RNAi cell line. FLAM3 was previously reported as a FAZ flagellum domain protein required for FAZ elongation and cell morphogenesis (13, 29). However, unlike its interacting partner proteins ClpGM6 and FAZ27, the localization of FLAM3 extends beyond the FAZ flagellum domain to the unattached flagellum region but does not reach the flagellar tip in uniflagellated cells (29). In biflagellated cells, FLAM3 is additionally localized to the FC (13). We confirmed this localization pattern of FLAM3 in noninduced control cells (Fig. 6*A*). In the uniflagellated 1N1K cells, knockdown of cAMP-BP1 reduced the average FLAM3 length by ∼17% from ∼13.5 μm to ∼11.2 μm (Fig. 6, *A* and *B*), and the FLAM3 occupancy of the flagellum was reduced by 19% from ∼94% to ∼75% (Fig. 6*C*, top left). cAMP-BP1 knockdown significantly reduced the FAZ length (Fig. 6, *A* and *B*), and the FAZ occupancy of the flagellum was reduced by ∼30% from ∼76% to ∼46% (Fig. 6*C*, top right). The average length of the flagellum was slightly but insignificantly increased (Fig. 6*B*), but the average length of the free flagellum was significantly increased (Fig. 6*B*). The average length of the FLAM3 in the free flagellum was increased by ∼69% from ∼2.6 μm to ∼4.4 μm (Fig. 6*B*), and the FLAM3 occupancy of the free flagellum was reduced by ∼14% from ∼70% to ∼56% (Fig. 6*C*, bottom left). Strikingly, the ratio of FLAM3 in the free flagellum and FLAM3 in the attached part of the flagellum was increased from an average of ∼0.25 to ∼ 0.71 (Fig. 6*C*, bottom right). Overall, cAMP-BP1 knockdown caused a slight but insignificant decrease of the overall FLAM3 length within the entire flagellum, but a significant increase of the FLAM3 length in the free flagellum (Fig. 6, *B* and *C*). In the biflagellated 2N2K cells, cAMP-BP1 knockdown caused the reduction of FLAM3 length in the new flagellum, albeit it was still longer than the intracellular FAZ filament (Fig. 6*A*). Notably, in all the biflagellated cells (>1000) examined, FLAM3 was no longer detectable at the FC (Fig. 6*A*), suggesting that cAMP-BP1 knockdown disrupted the localization of FLAM3 to the FC.

The results presented above suggest that cAMP-BP1 knockdown exerted different effects on FLAM3 and the FAZ1-labeled intracellular FAZ filament. However, it remains unclear whether the effect is specific to FLAM3 or is general to other FAZ flagellum domain proteins. To differentiate between a specific effect on FLAM3 and a general effect on FAZ flagellum domain proteins, we further examined the effect of cAMP-BP1 knockdown on the FAZ flagellum domain proteins ClpGM6 (12) and FAZ27 (14), both of which interacts with FLAM3, despite that both proteins are exclusively localized to the FAZ flagellum domain (12, 13, 14). Knockdown of cAMP-BP1 reduced the ClpGM6 length and the CC2D length to a similar extent (Fig. 6*D*), resulting in almost identical length for the ClpGM6-labeled FAZ flagellum domain and the CC2D-labeled intracellular FAZ filament. Similar effects were observed for cAMP-BP1 knockdown on FAZ27 (Fig. 6*E*). Thus, knockdown of cAMP-BP1 appears to exert a specific effect on FLAM3, by disrupting its assembly in the free flagellum and impairing its localization to the FC. In contrast, cAMP-BP1 knockdown appears to cause a general effect on the FAZ, by disrupting the elongation of the FAZ flagellum domain and the intracellular FAZ filament.

### Determination of the structural motifs in cAMP-BP1 required for cAMP-BP1 function

**Figure 7:**
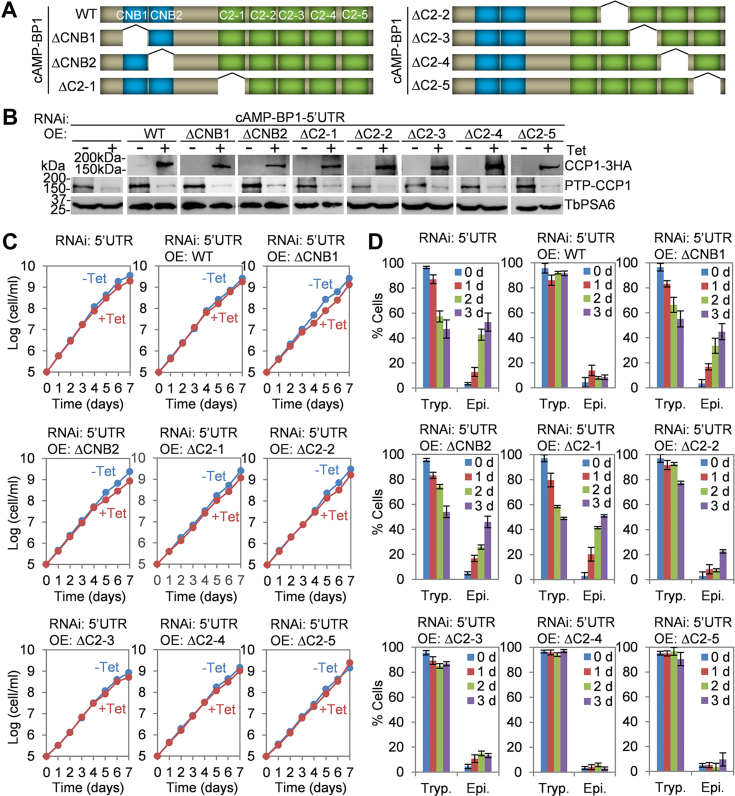
**Determination of the structural motifs required for cAMP-BP1 function**. *A*, schematic drawing of the WT and deletion mutants of the cAMP-BP1 protein used for complementation. *B*, Western blotting to monitor the knockdown of cAMP-BP1, which was endogenously tagged with an N-terminal PTP epitope, and the ectopic OE of WT and truncation mutants of cAMP-BP1, which was tagged with a C-terminal triple HA epitope. TbPSA6 served as a loading control. *C*, cell growth curves of cAMP-BP1-5′UTR RNAi cell line and its complementation cell lines induced with or without tetracycline. *D*, effect of cAMP-BP1-5′UTR RNAi and the complementation with WT and truncation mutants of cAMP-BP1 on cell morphogenesis. RNAi and ectopic overexpression were induced for up to 3 days. Error bars indicate S.D. from three independent experiments. OE, overexpression; RNAi, RNA interference.

cAMP-BP1 possesses two putative CNB domains at the N terminus and five C2-domains at the C terminus (Fig. 1). The presence of CNB domains in cAMP-BP1 implies potential cAMP-binding capability and potential regulation by cAMP. However, when we tried to perform *in vitro* cAMP binding assays using the commercially available cAMP-agarose beads (Sigma-Aldrich, Cat#: A0144; BIOLOG, Cat#: A054), both beads showed nonspecific binding to proteins tagged and expressed in *T. brucei* and in bacteria (data not shown). Therefore, we were unable to experimentally test whether cAMP-BP1 is a cAMP-binding protein. We instead tested whether these CNB domains are required for cAMP-BP1 function *in vivo* in trypanosomes. To this end, we generated a new cAMP-BP1 RNAi cell line by targeting its 5′UTR (the *cAMP-BP1* gene has no 3′UTR sequence but a 618-bp of 5′UTR sequence, see Tb927.10.5240 in the TriTrypDB database) and then ectopically expressed the WT or the truncation mutants of the cAMP-BP1 protein lacking each of the two CNB domains and the five C2 domains (Fig. 7*A*).

Induction of cAMP-BP1-5′UTR RNAi resulted in the knockdown of cAMP-BP1, which was endogenously tagged with a C-terminal triple HA epitope (Fig. 7*B*) and caused a weak growth defect (Fig. 7*C*), similar to that caused by cAMP-BP1 RNAi (Fig. 2*B*). Knockdown of cAMP-BP1 by targeting its 5′UTR also resulted in an increase of epimastigote-like 1N1K cells to ∼53% of the total 1N1K cell population after 3 days (Fig. 7*D*). Thus, cAMP-BP1-5′UTR RNAi recapitulated the phenotypes caused by cAMP-BP1 RNAi, which targets the coding region of the *cAMP-BP1* gene (Fig. 2).

We next ectopically expressed full-length cAMP-BP1 and multiple cAMP-BP1 truncation mutants in the cAMP-BP1-5′UTR RNAi cell line. RNAi and ectopic expression of the full-length or the truncation mutants of cAMP-BP1 were induced by tetracycline and confirmed by Western blotting (Fig. 7*B*). The expression level of the ectopically expressed cAMP-BP1 and its truncation mutants is close to that of the endogenous cAMP-BP1 protein. While expression of WT cAMP-BP1 rescued the growth defects of the cAMP-BP1-5′UTR RNAi cells, as expected, expression of three C2 domain-deletion mutants, ΔC2-3, ΔC2-4, and ΔC2-5, also rescued the growth defects (Fig. 7*C*). Quantitation of the 1N1K cells with either trypomastigote morphology or epimastigote-like morphology showed no significant accumulation of epimastigote-like cells in the complementation cell lines expressing ectopic WT cAMP-BP1 or each of these three C2 domain-deletion mutants (Fig. 7*D*). These results suggest that these three C2 domains are not required for cAMP-BP1 function. Expression of each of the two CNB domain-deletion mutants, ΔCNB1 and ΔCNB2, or the C2 domain #1-deletion mutant, ΔC2-1, was unable to rescue the weak growth defects of CAMP-BP1-5′UTR RNAi cells (Fig. 7*C*), and quantitation of the morphology of the 1N1K cells in these three complementation cell lines showed an accumulation of epimastigote-like cells to ∼45 to 51% of the 1N1K cell population after 3 days (Fig. 7*D*). These results suggest that the two CNB domains and the C2 domain #1 are each required for cAMP-BP1 function. Finally, expression of the C2 domain #2-deletion mutant, ΔC2-2, also was unable to rescue the weak growth defects of cAMP-BP1-5′UTR RNAi cells (Fig. 7*C*), but there was only a moderate accumulation of epimastigote-like 1N1K cells to ∼23% after 3 days (Fig. 7*D*). It appears that deletion of the C2-2 domain partially disrupts cAMP-BP1 function.

## Discussion

Major morphology changes during the life cycle form transition in trypanosomes appear to involve the modulation of the FAZ, as depletion of three FAZ flagellum domain proteins, ClpGM6 (12), FLAM3 (13, 29), and FAZ27 (14), two intracellular FAZ filament proteins, FAZ9 (7) and TbSAS-4 (15), and a flagellar tip-enriched orphan kinesin, KIN-E (30), in the trypomastigote form of *T. brucei* generates epimastigote-like cells. In this study, we identified a novel FC- and new FAZ tip-localized protein named cAMP-BP1, whose knockdown in the trypomastigote form of *T. brucei* also generated epimastigote-like cells (Figs. 2 and 3). The morphological differences between the trypomastigote form and the epimastigote form include the length of the FAZ, the length of the unattached flagellum, and the position of the kinetoplast and the flagellum relative to the nucleus (11). The trypomastigote form has a short unattached flagellum, a long FAZ structure, and posterior positioning of the kinetoplast and the flagellum, whereas the epimastigote form has a long unattached flagellum, a short FAZ structure, and anterior positioning of the kinetoplast and the flagellum. Knockdown of each of the above-mentioned proteins disrupted the elongation of the FAZ structure, by impairing either the FAZ flagellum domain or the intracellular FAZ filament (7, 12, 13, 14, 15, 29, 30). Because FAZ elongation appears to control basal body positioning (31) and, hence, flagellum positioning, it is likely that the primary defects caused by cAMP-BP1 knockdown are on the elongation of the FAZ structure, which consequently led to defective positioning of the flagellum and the basal body-associated kinetoplast, generating cells with an epimastigote-like morphology. These findings suggest that cAMP-BP1 acts to maintain the typical trypomastigote morphology by promoting FAZ elongation and flagellum positioning.

Knockdown of cAMP-BP1 appears to exert a specific effect on the assembly of FLAM3 and a general effect on the assembly of other FAZ flagellum domain proteins, such as ClpGM6 and FAZ27, and the intracellular FAZ filament proteins, such as CC2D and FAZ1 (Fig. 6). FLAM3 was previously suggested to be a FAZ flagellum domain component, and it interacts with ClpGM6 (13) and FAZ27 (14), but its localization pattern differs from ClpGM6 and FAZ27 by extending beyond the distal tip of the FAZ and by additionally concentrating at the FC (13, 29), likely to reside in the FC zone 1 or zone 2 within the new flagellum. Thus, complex formation between these proteins likely occurs within the FAZ flagellum domain, but not in the unattached flagellum region and the FC. It was previously hypothesized that FLAM3 in the unattached flagellum region might be the excess proteins unable to assemble into the FAZ flagellum domain (13). In this regard, it seems that more FLAM3 proteins were unable to assemble into the FAZ flagellum domain in cAMP-BP1 RNAi cells, because the length of FLAM3 in the unattached flagellum became longer in cAMP-BP1 RNAi cells than in control cells (Fig. 6, *A* and *B*). This is plausible, as the FAZ flagellum domain was shorter in cAMP-BP1 RNAi cells and likely contained less assembled proteins. Another possibility is that FLAM3 in the unattached flagellum region has unidentified functions, and its assembly in this region is independent of the FAZ flagellum domain. In contrast to FLAM3, the length of the FAZ flagellum domain and the length of the intracellular FAZ filament were reduced to almost the same extent in cAMP-BP1 RNAi cells (Fig. 6, *C*–*E*). This suggests a general effect exerted by cAMP-BP1 knockdown on the assembly of the FAZ structure as a whole.

The inhibition of FAZ elongation by cAMP-BP1 knockdown suggests that cAMP-BP1 plays a role in promoting FAZ assembly/elongation. Assembly of the FAZ occurs at its proximal end, where new components are incorporated into the elongating FAZ (9, 10), in contrast to the assembly of the flagellum, which occurs at its distal tip (32). The localization of cAMP-BP1 to the distal end of the elongating FAZ and the FC (Fig. 1*E*) raises an interesting question about how cAMP-BP1 promotes FAZ elongation at the location that is opposite to its site of assembly. The underlying mechanisms for the assembly of the FAZ remain elusive, but two models, a “push” model and a “pull” model, have been proposed to explain the assembly mechanisms of the FAZ (8). In the “push” model, the incorporation of new FAZ components at the proximal end could “push” the assembled FAZ structure toward the distal direction, as the flagellum elongates at its distal end. In the “pull” model, a structure connecting the distal tips of the flagellum and the FAZ could “pull” the FAZ toward the distal direction for its components to be incorporated at the proximal end (8). It appears that cAMP-BP1 localized at the new FAZ tip and the FC (or the flagellar tip) could serve to connect the two structures for the assembly of the FAZ through the “pull” mechanism. In the absence of cAMP-BP1, the connection between the FAZ tip and the flagellar tip is disrupted; therefore, there is no pulling force from the tip of the elongating flagellum that can facilitate FAZ assembly, thereby resulting in defective assembly/elongation of the FAZ.

At the FC, cAMP-BP1 and FLAM3 are interdependent for their localization (Figs. 5*C* and 6*A*) and they reside in such a close proximity that they may interact or are in a larger protein complex (Fig. 5*B*). One role for cAMP-BP1 at the FC apparently is to maintain flagellar connection (Fig. 4), but whether FLAM3 at the FC plays a similar role in maintaining flagellar connection was not investigated previously (13, 29). Therefore, cAMP-BP1 apparently functions as a typical FC component, like other FC proteins characterized previously (4, 7). Depletion of individual FC components from different FC subdomains disrupts flagellar connection, but does not affect cell proliferation (4, 7). It is thus possible that depletion of cAMP-BP1 from the FC also does not affect cell proliferation, whereas the weak growth defect of cAMP-BP1 RNAi cells (Fig. 2) may be attributed to the depletion of cAMP-BP1 from the new FAZ tip, by which it impaired the elongation/assembly of the new FAZ in biflagellated cells (Fig. 3, *C* and *D*). These biflagellated cells with a short new FAZ apparently were able to proceed through cytokinesis to generate uniflagellated daughter cells with a short FAZ and an epimastigote-like morphology (Figs. 2, *D*–*F* and 3, *A* and *B*). The length of the new FAZ in a biflagellated cell determines the cell division plane in trypanosomes (8), and the minimum length of the new FAZ required for cytokinesis was estimated to be 3 μm in a previous study (13). After cAMP-BP1 RNAi induction for 48 h, ∼10% of the biflagellated cells contained a new FAZ that was shorter than 3 μm (Fig. 3*D*). Thus, the majority of the biflagellated cells from the cAMP-BP1 RNAi-induced population was able to proliferate, as the length of their new FAZ remained above the threshold of length required for cytokinesis. The weak growth defects of the cAMP-BP1 RNAi cells (Fig. 2*B*) and the accumulation of xNyK cells after cAMP-BP1 RNAi (Fig. 2*C*) were attributed to the inhibited cytokinesis of those biflagellated cells with the new FAZ below the threshold of length required for cytokinesis.

An intriguing structural feature of cAMP-BP1 is the presence of two putative CNB domains (CNB1 and CNB2) that are each required for cAMP-BP1 function (Fig. 7). Both domains adopt the typical fold of a cyclic nucleotide-binding domain found in protein kinase A (Fig. S1*A*), despite that CNB2 lacks the conserved arginine residue (Fig. S1*B*), which, in the known cAMP-binding proteins, binds the exocyclic phosphate of cAMP (20). The CNB1 domain in cAMP-BP1, however, contains all the structural elements required for cAMP binding (Fig. S1); thus, presumably it should be able to bind cAMP. Nonetheless, whether the two CNB domains are capable of binding cAMP remains to be experimentally investigated. There are two possibilities to explain the essentiality of CNB2 for cAMP-BP1 function (Fig. 7). One is that CNB2 is able to bind cAMP and this cAMP-binding capability does not require the arginine residue. In this scenario, deletion of CNB2 disrupts cAMP-BP1 function by impairing its full cAMP-binding capability. The other possibility is that CNB2 is unable to bind cAMP due to the lack of the conserved arginine residue. In this scenario, deletion of CNB2 may disrupt the folding of cAMP-BP1, causing loss of function.

In summary, we have identified a putative cAMP-binding protein that localizes to the new FAZ tip and the FC, the first of such proteins with dual localization at these cytoskeletal structures. We further demonstrated that this protein promotes cell morphogenesis by controlling the assembly/elongation of the new FAZ to maintain the positioning of the flagellum and its associated kinetoplast and cytoskeletal structures and facilitates flagellar connection by maintaining FC integrity. The identification of a putative cAMP-binding protein as a regulator of cell morphogenesis and flagellar connection implies the potential involvement of cAMP signaling in controlling these processes during trypanosome life cycle transitions.

## Experimental procedures

### Trypanosome cell culture and RNAi

The procyclic form of *T. brucei* Lister427 strain and 29-13 strain (33) were grown in the SDM-79 medium containing 10% heat-inactivated fetal bovine serum (Atlanta Biologicals, Inc) at 27 °C and in the SDM-79 medium supplemented with 10% heat-inactivated fetal bovine serum, 15 μg/ml G418, and 50 μg/ml hygromycin at 27 °C, respectively. Cells were diluted with fresh medium every 3 to 4 days or when the cell density reached 5 × 10^6^/ml.

RNAi was performed essentially according to our published procedures (34). To generate the cAMP-BP1 RNAi cell line, a 582-bp DNA fragment (nucleotides 1–582) from the coding region of the *cAMP-BP1* gene was cloned into the pZJM vector (35), and the resulting plasmid pZJM-cAMP-BP1 was linearized with NotI and used to transfect the 29-13 strain by electroporation. Transfectants were selected with 2.5 μg/ml phleomycin, and cloned by limiting dilution in a 96-well plate containing the SDM79 medium supplemented with 20% fetal bovine serum and appropriate antibiotics. The FLAM3 RNAi cell line was reported previously (30). Induction of RNAi was performed by incubating the RNAi cell lines with 1.0 μg/ml tetracycline, and cell proliferation was monitored daily by cell counting with a hemacytometer. Three independent clonal RNAi cell lines were selected and analyzed, which generated similar phenotypes, so only the results obtained from the characterization of one clonal cell line were presented in this work.

### Complementation of cAMP-BP1 RNAi with WT and domain-deletion mutants of cAMP-BP1

For cAMP-BP1 RNAi complementation with cAMP-BP1 and its domain-deletion mutants, a new cAMP-BP1 RNAi cell line was generated by targeting against the 5′UTR of the *cAMP-BP1* gene. A 618-bp sequence of the 5′UTR of *cAMP-BP1* gene, which does not overlap with the gene upstream of *cAMP-BP1* on the chromosome, was cloned into the pZJM-PAC vector, and the resulting construct, pZJM-cAMP-BP1-5′UTR, was linearized and used to transfect the 29-13 strain by electroporation. Transfectants were selected with 1 μg/ml puromycin, and cloned by limiting dilution in a 96-well plate as described above. Three clonal cell lines were selected and characterized by verifying the knockdown efficiency and the phenotypes, and one clonal cell line was used for complementation. The DNA sequences encoding the full-length cAMP-BP1 protein or the cAMP-BP1 domain-deletion mutant proteins were each cloned into the pLew100-3HA-BLE vector, and the resulting plasmids were linearized by NotI restriction digestion and used to transfect the cAMP-BP1-5′UTR RNAi cell line. Transfectants were selected with 2.5 μg/ml phleomycin in addition to 1 μg/ml puromycin, 15 μg/ml G418, and 50 μg/ml hygromycin B, and cloned by limiting dilution in a 96-well plate as described above. Three clonal cell lines were selected and characterized to verify the ectopic expression of the 3HA-tagged cAMP-BP1 and its domain-deletion mutants, and one clonal cell line was used for detailed characterization and data presentation.

### *In situ* epitope tagging of proteins

Epitope tagging of cAMP-BP1, FLAM3 (29), ClpGM6 (12), FAZ27 (14), and FC1 (7) from their respective endogenous locus was carried out using the PCR-based epitope tagging method (36). For colocalization of cAMP-BP1 with other proteins, including FLAM3, FC1, FCP2, FCP3, and FCP4, cAMP-BP1 was tagged with a C-terminal triple HA epitope (puromycin resistance) and the other proteins were each tagged with an N- or C-terminal PTP epitope (blasticidin resistance) in the *T. brucei* Lister427 strain. Successful transfectants were selected with 10 μg/ml blasticidin and 1 μg/ml puromycin and further cloned by limiting dilution in a 96-well plate.

cAMP-BP1 was also tagged with a C-terminal triple HA epitope (puromycin resistance) in the cAMP-BP1 RNAi cell line and the FLAM3 RNAi cell line, and ClpGM6 and FAZ27 were each tagged with an N-terminal and C-terminal triple HA epitope (puromycin resistance), respectively, in the cAMP-BP1 RNAi cell line. Transfectants were selected with 1 μg/ml puromycin and further cloned by limiting dilution in a 96-well plate. FLAM3 was tagged with an N-terminal PTP epitope (blasticidin resistance) in the cAMP-BP1 RNAi cell line. Transfectants were selected with 10 μg/ml blasticidin and further cloned by limiting dilution in a 96-well plate.

### Immunofluorescence microscopy

Trypanosome cells were adhered onto the coverslips, fixed with cold methanol for 30 min at −20 ^o^C, and blocked with 3% bovine serum albumin in PBS for 30 min at room temperature. Cells on the coverslips were incubated with the following primary antibodies, anti-HA monoclonal antibody (1:400 dilution, Sigma-Aldrich), anti-Protein A polyclonal antibody (1:400 dilution, Sigma-Aldrich), anti-CC2D polyclonal antibody (1:1000 dilution) (37), anti-PFR2 monoclonal antibody (clone L8C4, 1:1 dilution) (38), and anti-FAZ1 monoclonal antibody (clone L3B2, 1: 10 dilution) (38), for 60 min at room temperature. Cells were washed three times with PBS and incubated with FITC-conjugated anti-mouse IgG (1:400 dilution, Sigma-Aldrich) or Cy3-conjugated anti-rabbit IgG (1:400 dilution, Sigma-Aldrich) for 60 min at room temperature. Cells on the coverslips were washed three times with PBS, mounted with 4′,6-diamidino-2-phenylindole-containing VectaShield mounting medium (Vector Labs), and imaged under a fluorescence microscope. Images were acquired using the Slidebook software (https://www.intelligent-imaging.com/slidebook) and processed with Photoshop.

### Scanning and transmission electron microscopy

Scanning electron microscopy was performed according to our published procedures (22, 23). Trypanosome cells were fixed with 2.5% (v/v) glutaraldehyde in the cell culture medium for 2 h at room temperature and then washed with PBS. Cells were adhered on glass coverslips and washed three times with deionized water before being dehydrated in a series of alcohol (30%, 50%, 70%, 90%, and 100%) for 10 min each at room temperature. Cells on the coverslip were dried by critical point drying and then were coated with a 5-nm metal film (Pt:Pd 80:20, Ted Pella Inc.) using a sputter-coater (Cressington Sputter Coater 208 HR, Ted Pella Inc). Cells were imaged using Nova NanoSEM 230 (FEI) with the following settings: the scanning work distance set at 5 mm and the accelerating high voltage set at 8 kV.

Transmission electron microscopy was performed essentially as described previously (39). Whole-mount cytoskeletons of trypanosome cells were prepared by washing cells twice with PBS, settling onto freshly charged carbon and Formvar-coated grids, and incubating with the PIPES-EGTA-MgSO_4_-EDTA buffer (100 mM PIPES, pH 6.9, 2 mM EGTA, 1 mM MgSO_4_, 0.1 mM EDTA) containing 1% Nonidet P-40 for 5 min. Cytoskeletons on the grids were then fixed with glutaraldehyde for 30 min and negatively stained with aurothioglucose in distilled water. Grids were imaged using a JEOL 1400 TEM at 120 kV and captured with a Gatan charge-coupled device camera.

### Proximity ligation assay

PLA was carried out to detect the *in vivo* interaction between cAMP-BP1 and FLAM3 using the Duolink *In Situ* PLA Detection kit (Cat#: DUO92008, Sigma-Aldrich) according to the manufacturer’s instructions. The *T. brucei* Lister427 cells coexpressing cAMP-BP1-3HA and PTP-FLAM3 from their respective endogenous locus and expressing cAMP-BP1-3HA or PTP-FLAM3 from their respective endogenous locus were adhered onto glass coverslips and fixed with cold methanol for 30 min at −20 °C. Cells on the coverslip were first blocked with the Duolink blocking solution at room temperature and then incubated with the anti-Protein A antibody and the anti-HA antibody for 60 min at room temperature. Subsequently, cells were washed with Buffer A and probed with the Duolink *In Situ* PLA Probe anti-Mouse MINUS (#DUO92004, Sigma-Aldrich) and the Duolink *In Situ* PLA Probe anti-Rabbit PLUS (#DUO92002, Sigma-Aldrich) for 60 min at room temperature. Cells were washed with Buffer A, and incubated with the ligation solution and then the amplification solution in a humidity chamber at 37 °C for 100 min. Cells were washed with Buffer B, mounted in the Duolink *In Situ* Mounting Medium, and imaged under a fluorescence microscope. For control experiments, cells were incubated either with buffer only or with only one antibody (anti-Protein A antibody or anti-HA antibody), following the same procedures.

### Data analysis and statistical analysis

The ImageJ software (National Institutes of Health, Bethesda, MD; http://imagej.nih.gov/ij/) was used for measurement of FAZ length, flagellum length, cell body length, and free flagellum length, kinetoplast-to-nucleus distance, kinetoplast-to-posterior distance, and the length of the FLAM3 signal. Statistical analysis was conducted using the two-tailed Student’s *t* test or one-way ANOVA. Error bars represented SD from three independent experiments.

To avoid bias, samples were blinded to the investigator who took images under the microscope, and counting of cells in the images was carried out by another investigator.

## Data availability

All data are contained within the manuscript.

## Supporting information

This article contains supporting information.

## Conflict of interest

The authors declare that they have no conflicts of interest with the contents of this article.
