# Supplementary material for: A putative cAMP-binding protein in Trypanosoma brucei cooperates with FLAM3 to promote flagellar connection and cell morphogenesis
Source: J Biol Chem. 2024 Oct 5;300(11):107856. doi: 10.1016/j.jbc.2024.107856 (PMC11555346; doi:10.1016/j.jbc.2024.107856)
Supplement: Supporting information [file mmc1.pdf]

Supporting Information

Title: A putative cAMP-binding protein in Trypanosoma brucei cooperates with FLAM3 to promote flagellar connection and cell morphogenesis

Authors: Qing Zhou, Phu Van Nguyen, and Ziyin Li

Figures S1-S3

Figure S1

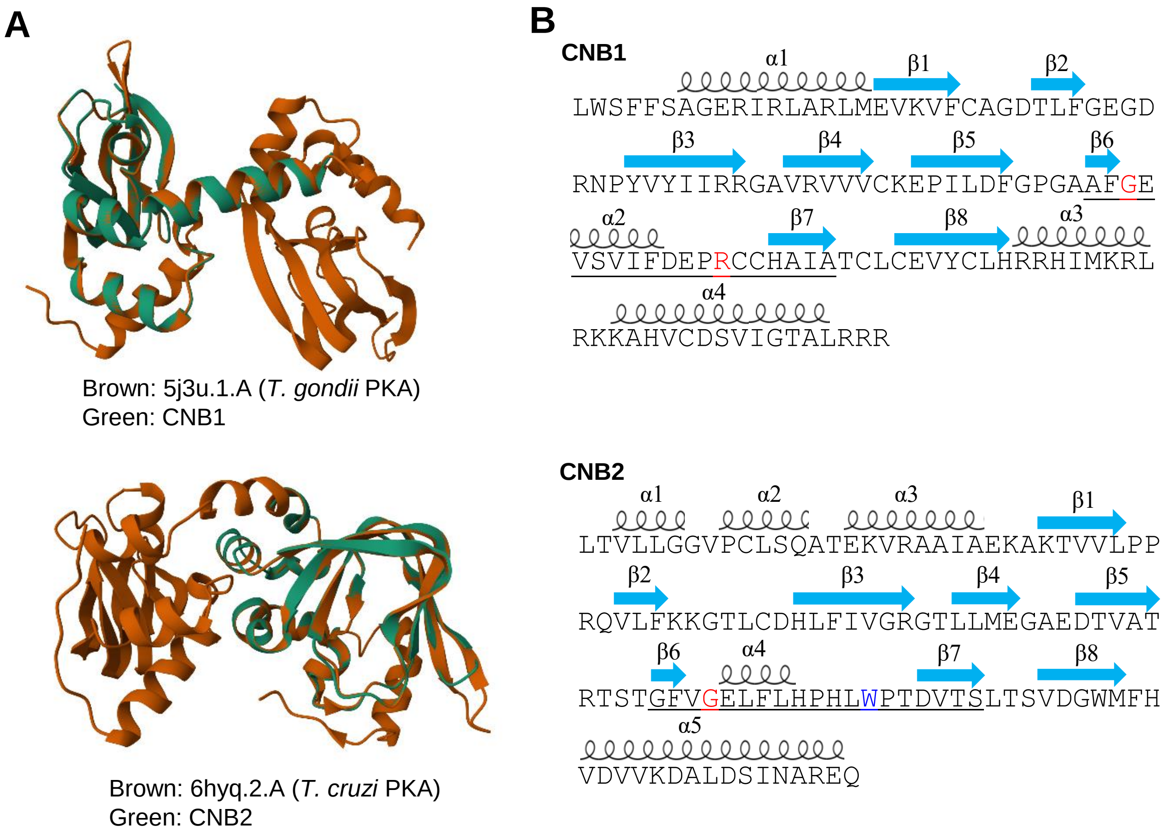

Figure S1. Structural analysis of the CNB domains in cAMP-BP1. (**A**). Structural alignment of CNB1 and 5j3u.1.A, and CNB2 and 6hyq.2.A. (**B**). The α-helices and the β-strands in CNB1 and CNB2 of cAMP-BP1 were determined according to the structural modeling using SWISS-MODEL. The underlined residues indicate the predicted PBC. The residues highlighted in red indicate the conserved glutamate and arginine residues in CNB1 and the conserved glutamate residue in CNB2, whereas the tryptophan residue highlighted in blue in CNB2 indicates that the conserved arginine residue has been replaced by tryptophan.

Figure S2

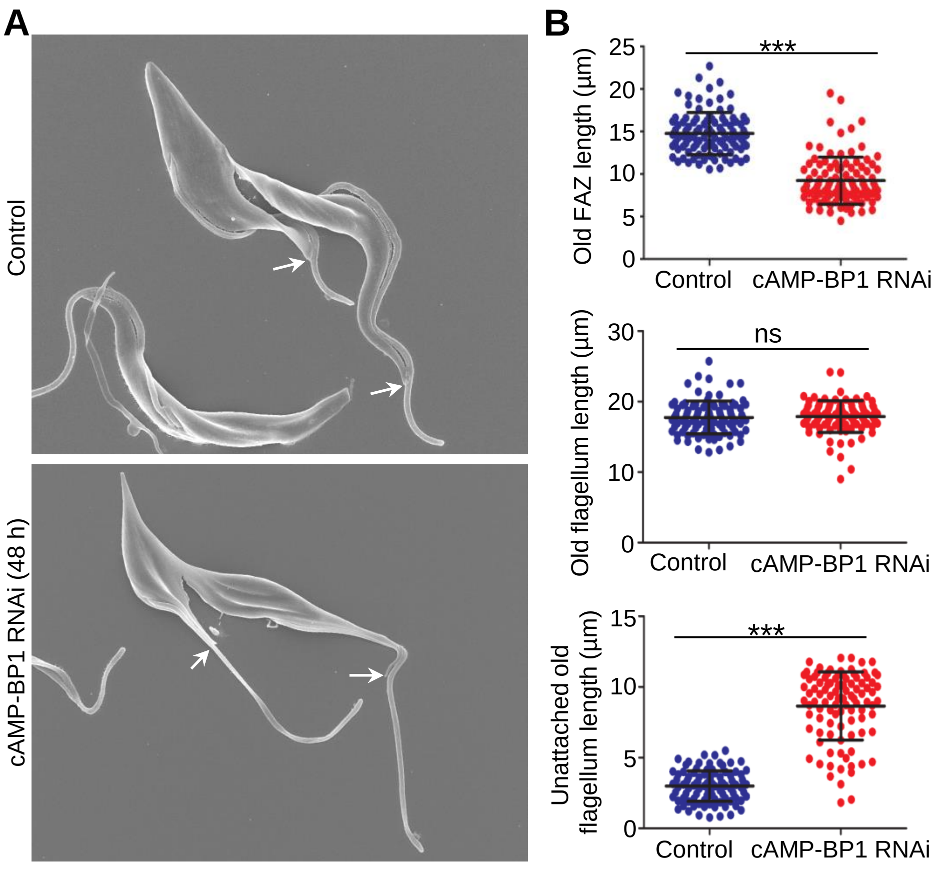

Figure S2. cAMP-BP1 RNAi generates bi-flagellated cells with long, unattached new and old flagella. (**A**). Scanning electron microscopy of bi-flagellated cells from control and cAMP-BP1 RNAi-induced cell population. Arrows indicate the distal tip of the cell body. Scale bar: 5 µm. (**B**). Morphometric measurement of 2N2K cells collected from control and cAMP-BP1 RNAi-induced population. The length of the old FAZ filament, the old flagellum, and the unattached old flagellum was measured and plotted (n=100). ***: p<0.001; ns: no significance.

Figure S3

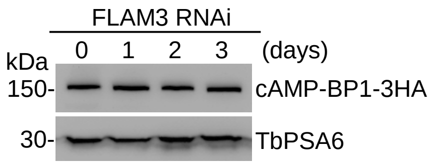

Figure S3. Effect of FLAM3 knockdown on the level of cAMP-BP1 protein. cAMP-BP1 was endogenously tagged with a triple HA epitope and detected by the anti-HA antibody. TbPSA6 served as a loading control.
